# Perioperative optimisation in low- and middle-income countries (LMICs): A systematic review and meta-analysis of enhanced recovery after surgery (ERAS)

**DOI:** 10.7189/jogh.13.04114

**Published:** 2023-10-03

**Authors:** Aya M Riad, Aisling Barry, Stephen R Knight, Carlie J Arbaugh, Parvez D Haque, Thomas G Weiser, Ewen M Harrison

**Affiliations:** 1 Royal Infirmary of Edinburgh, Edinburgh, United Kingdom; 2 Centre for Medical Informatics, Usher Institute Edinburgh, UK; 3 Department of Surgery, Stanford Health Care and Stanford University, Stanford, California, USA; 4 Department of General Surgery, Christian Medical College and Hospital, Punjab, India

## Abstract

**Background:**

Enhanced recovery after surgery (ERAS) protocols have largely been incorporated into practice in high-income settings due to proven improvement in perioperative outcomes. We aimed to review the implementation of ERAS protocols and other perioperative optimisation strategies in low- and middle-income countries (LMICs) and their impact on length of hospital stay (LOS).

**Methods:**

We searched MEDLINE, PubMed, Global Health (CABI), WHO Global Index Medicus, Index Medicus, and Latin American and Caribbean Health Sciences Literature (LILACS) for studies incorporating ERAS or other prehabilitation approaches in LMICs. We conducted a pooled analysis of LOS using a random-effects model to evaluate the impact of such programs. This systematic review was pre-registered on PROSPERO.

**Results:**

We screened 1205 studies and included 70 for a full-text review; six were eligible for inclusion and five for quantitative analysis, two of which were randomised controlled trials. ERAS was compared to routine practice in all included studies, while none implemented prehabilitation or other preoperative optimisation strategies. Pooled analysis of 290 patients showed reduced LOS in the ERAS group with a standardised mean difference of -2.18 (95% confidence interval (CI) = -4.13, -.0.05, *P* < 0*.*01). The prediction interval was wide (95% CI = -7.85, 3.48) with substantial heterogeneity (*I*^2^ = 94%).

**Conclusions:**

Perioperative optimisation is feasible in LMICs and appears to reduce LOS, despite high levels of between-study heterogeneity. There is a need for high-quality data on perioperative practice in LMICs and supplementary qualitative analysis to further understand barriers to perioperative optimisation implementation.

**Registration:**

PROSPERO: CRD42021279053.

Surgery is a physiologically demanding event [1,2], and modifying aspects of the endocrine stress response and postoperative catabolic processes to optimise care in the perioperative period is increasingly being recognised as a means of improving outcomes [3]. This began with the concept of fast track surgery [4], followed by enhanced recovery after surgery (ERAS) [5] which described pre-, intra-, and post-operative factors to improve patient outcomes after surgery. There has recently been increased interest in prehabilitation [6], multimodal exercise, and nutritional and psychological interventions [7,8] aiming to better prepare patients for surgery. Prehabilitation, preoperative nutritional care, and preoperative optimisation (including optimising comorbid medical conditions and alcohol and smoking cessation) are now incorporated in the 2018 ERAS Recommendations for elective colorectal surgery [9].

There is little doubts about the benefits of perioperative optimisation protocols [10], yet their feasibility and benefits in low- and middle-income countries (LMICs) remain unexplored [11]. The Lancet Commission on Global Surgery [12] highlighted the great inequity in surgical care provision in LMICs, with nearly five billion people still lacking access to safe and affordable surgical care [13]. Large multicentre studies such as GlobalSurg 3 [14] and the African Surgical Outcomes Study (ASOS) [15] have shown postoperative mortality to be significantly worse in LMICs, with a proportion of these deaths attributed to a reduced “capacity to rescue” patients from complications in the postoperative period [16]. Thus, an intervention such as ERAS, which has been cost-effective in high-income settings [17] and is focused on increasing the physiological reserve of patients and reducing postoperative complications, theoretically could be significantly beneficial in LMICs.

There is a need for increased uptake of validated perioperative optimisation protocols in LMICs [18,19], further shown by the anticipated release of LMIC-specific ERAS guidelines [9]. However, considerations for how they may be adapted and implemented are lacking. We aimed to determine the feasibility of perioperative optimisation for surgery in LMICs and its impact on postoperative length of hospital stay (LOS).

## METHODS

### Search strategy

We searched MEDLINE, PubMed, Global Health (CABI), WHO Global Index Medicus, Index Medicus (WPRIM for the Western Pacific and IMSAR for the South Asian region) and Latin American and Caribbean Health Sciences Literature (LILACS) for randomised controlled trials (RCTs) and prospective or retrospective cohort studies conducted in lower middle- or low-income countries that compared perioperative interventions, comprising either aspects of the ERAS pathway or prehabilitation to routine care. We defined LMIC status according to World Bank Analytical Classification based on gross national income (GNI) per capita at the time the search was undertaken (23 December 2021) [20]. We used Cochrane LMIC filters [21] in non-LMIC databases (MEDLINE and PubMed). Additionally, we hand-searched references and citations of included studies and relevant reviews or editorials for additional eligible studies.

We excluded reviews, editorials, case reports, surveys, protocols, studies only reporting quality of life (QOL) outcomes without using validated patient-reported outcome measures (PROMs), and studies conducted in high- or upper-middle-income countries. We also excluded studies conducted on paediatric or non-surgical patients; for reference, we defined surgical patients as those undergoing a procedure requiring general or neuraxial anaesthesia and thus excluded patients only undergoing endoscopy. There were no limits placed on date or language. We registered this systematic review on the International Prospective Register of Systematic Reviews (PROSPERO) (CRD42021279053) and performed it according to Preferred Reporting Items for Systematic Reviews and Meta-Analyses (PRISMA) guidelines [22] (Appendix 1 in the **Online Supplementary Document**).

### Study selection and data extraction

We managed the study selection process via Covidence (Covidence, Melbourne, Australia). Two reviewers (AMR and AB) independently screened all abstracts, while a third reviewer (SRK) who did not have access to the initial reviewers’ decisions resolved any discrepancies. LMIC status of all studies undergoing full-text review were checked against historical World Bank classifications. Two independent reviewers (AMR and AB) undertook full text review and risk of bias assessment.

Two reviewers (AMR and AB) piloted a data extraction template and made appropriate changes, after which they independently extracted data from each study using a standardised extraction form within the Covidence platform. We collected study details, intervention and ERAS elements applied, outcomes, and qualitative aspects of perceived benefits and adaptations required in a lower-income setting for each study (Appendix 2 in the **Online Supplementary Document**). The reviewers first attempted to resolve discrepancies through discussion; if unsuccessful, a third member of the study team (EMH) resolved the dispute.

### Quality assessment

We assessed quality and risk of bias using the Newcastle-Ottawa Scale for non-randomised and the Cochrane Risk of Bias tool for randomised studies [23,24]. Two reviewers (AMR and AB) independently assessed the quality of all studies with conflicts resolved by a third reviewer (EMH). We determined a priori that no studies would be excluded on the basis of low quality or surgical specialty and that we would not limit inclusion to randomised control trials due to the small numbers of available studies and similarities in ERAS protocol across specialties. However, we undertook a sensitivity analysis, including only general surgical patients to quantify the impact of this decision. The 2018 ERAS protocol for elective colorectal surgery [7] was used to compare elements implemented in all studies.

### Statistical analysis

Using the *meta* package in R, version 3.6.1. (R Core Team, Auckland, New Zealand) [22], we completed a meta-analysis of LOS, as this was the outcome reported most consistently amongst studies and the main outcome used in ERAS studies [10]. As we anticipated high levels of between-study heterogeneity (studies unlikely to be measuring the same underlying effect), we used a random-effects model with standardised mean differences. We used the restricted maximum likelihood estimator method [25] to calculate τ^2^ and Knapp-Hartung adjustments [26] for the pooled effect confidence interval. We also calculated a predication interval indicating the effect size future studies were likely to find. We considered a *P*-value of <0.05 to be statistically significant.

## RESULTS

**Figure 1:**
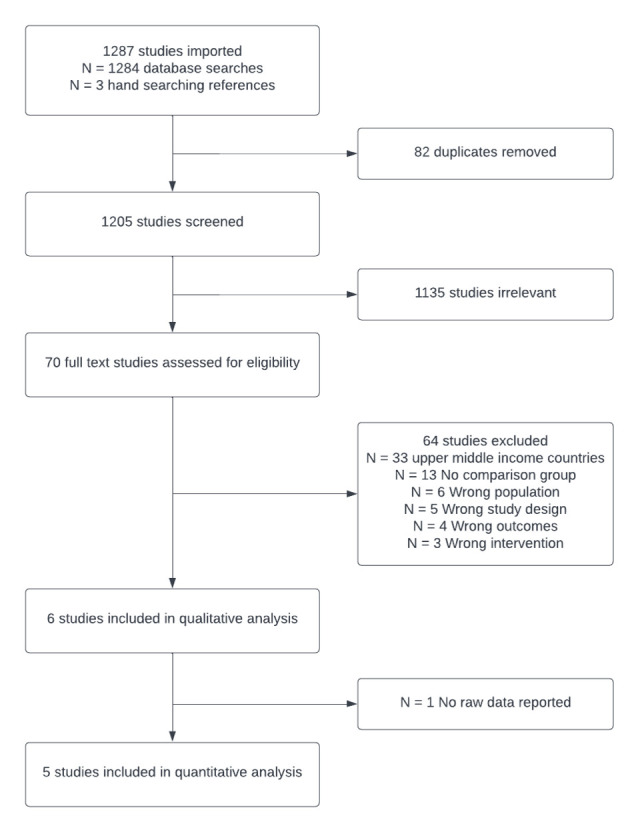
PRISMA flowchart.

We screened 1205 studies with 70 undergoing full text review (**Figure 1**); six were eligible for inclusion [27-32], with all except Gopakumar et al [30] reporting sufficient data to be included in quantitative analysis. The intervention in all studies was ERAS and the comparator was routine practice in the study hospitals. All studies included general surgical patients, except for Elayat et al. [32], which included neurosurgical patients. Two studies [29,31] exclusively included stoma reversal procedures. Only two studies [28,31] were RCTs. Three studies were conducted in India, which, alongside Egypt (where one study was based) was classified as a lower-middle-income country. Two studies were conducted in low income countries (Nepal and Pakistan) (**Table 1** and Appendix 3 in the **Online Supplementary Document**).

**Table 1:** Study characteristics

Study	Nanavati et al [27], 2014	Shetiwy et al [28], 2017	Kurmi et al [29], 2021	Gopakumar et al [30], 2020	Pirzada et al [31], 2017	Elayat et al [32], 2021
Country	India	Egypt	Nepal	India	Pakistan	India
Country income level at time of study	Lower middle income	Lower middle income	Low income	Lower middle income	Low income	Lower middle income
Speciality	General surgery	General surgery	General surgery	General surgery	General surgery	Neurosurgery
Number of patients, experimental /control	30/30	35/35	15/15	78/78	30/30	35/35
Cancer patients	Yes, some	Yes, all	Yes, some	Yes, some	No	Yes, all
Primary outcome	Length of stay	Length of stay	Seven-day readmission, length of stay, morbidity and mortality	Length of stay and morbidity	Length of stay	Length of ICU stay
Risk of Bias, tool: score	Newcastle-Ottawa: 5	Cochrane: some concerns	Newcastle-Ottawa: 4	Newcastle-Ottawa: 3	Cochrane: some concerns	Newcastle-Ottawa: 6
Inclusion criteria	Patients aged 16-66; elective gastrointestinal surgery with anastomosis distal to ileum	Patients with pathologically confirmed colorectal carcinoma; elective surgery, ASA I-III	Patients undergoing elective stoma closure, 16-70 y old; ASA I/II; BMI<30; live within two hours of hospital; access to phone and transport; next of kin staying with patient for 24 h	Patients undergoing elective abdominal surgerya aged <70 y	Patients undergoing two-end ileostomy reversal, aged >15	ASA I/II, aged 18 or above; patients with a single supratentorial space-occupying lesion eligible for elective craniotomy
Exclusion criteria	Patients undergoing emergency surgery; uncontrolled comorbid conditions	Previous abdominal surgery; patients undergoing emergency surgery; chronic pain syndrome	Emergency stoma closure; stoma closure by midline abdominal incision; uncontrolled comorbid conditions; previous or simultaneous abdominal surgery	Patients undergoing emergency surgery; moribund patients	Patients with neurological, renal or cardiac disease or diabetes; bed-ridden patients; patients on steroids; patients unable to understand commands	Patients undergoing emergency craniotomies; uncontrolled diabetes; patients with severe cognitive impairment who are unable to follow simple instructions

ICU – intensive care unit, ASA – American Society of Anaesthesiologists, BMI – body-mass index

### Patient population

The inclusion and exclusion criteria used by studies are summarized in **Table 1**. Most studies included cancer patients; two (Nanavati et al [27] and Pirzada et al [31]) noted the main reason for surgery being inflammatory bowel disease (IBD). Patients with uncontrolled comorbid conditions (e.g. diabetes or cardiac disease) were excluded from all studies apart from Shetiwy et al. [28] and Gopakumar et al. [30] . Additionally, Kurmi [29] required patients to have a body mass index (BMI) <30, to live within two hours of the hospital, and to have access to a phone, transport, and a responsible next of kin staying in the hospital with them for at least 24 hours (**Table 1**).

### Intervention

The intervention in all included studies was an ERAS protocol. There are currently no official ERAS guidelines in neurosurgery. Principles of an ERAS protocol for elective craniotomies were first suggested by Hagan et al. [33] and later adapted by study groups for use in trials [34], as was the case in Elayat et al. [32] (**Table 2**). All interventions took place exclusively in hospital settings, with the intervention duration being at most 48 hours preoperatively to discharge. Details of who was responsible for administering the intervention were unclear, as most studies focused on the surgeon and surgical trainees, as well as family members staying with patients. Only Elayat et al. [32] reported ERAS training for nursing and surgical trainees. Most studies did not report on compliance with the intervention; only Nanavati et al. [27] reported the number of patients who underwent each aspect of the protocol and Elayat et al. [32] described the use of an ERAS checklist for each patient to monitor compliance without reporting results.

**Table 2:** ERAS Protocol followed by studies

ERAS component*	Nanavati et al [27], 2014	Shetiwy et al [28], 2017	Kurmi et al [29], 2021	Gopakumar et al [30], 2020	Pirzada et al [31], 2017	Elayat et al [32], 2021
1. Preadmission counselling	Not reported	All patients	All patients	All patients	All patients	All patients
2. Preoperative optimisation	Not reported	Not reported	Not reported	All patients	Not reported	Not reported
3. Prehabilitation	Not reported	Not reported	Not reported	Not reported	Not reported	Not reported
4. Preoperative nutritional care	Not reported	Not reported	Not reported	Not reported	Not reported	Not reported
5. Management of anaemia	Not reported	Not reported	Not reported	Not reported	Not reported	Not reported
6. Prevention of nausea and vomiting	All patients	Not reported	Not reported	All patients	Not reported	Not reported
7. Selective pre-anaesthetic medication	Not reported	All patients	All patients	All patients	Not reported	Not reported
8. Antimicrobial prophylaxis and skin preparation	All patients	Not reported	Not reported	All patients	Not reported	Not reported
9. No bowel preparation	Some patients	Some patients	Not applicable	All patients	Not applicable	Not applicable
10. Maintaining preoperative euvolemia	Not reported	Not reported	Not reported	Not reported	Not reported	Not reported
11. No preoperative fasting and carbohydrate loading	All patients	All patients	All patients	All patients	All patients	All patients
12. Short term anaesthetic protocol & complete neuromuscular blockade reversal	Not reported	Not reported	Not reported	All patients	All patients	All patients
13. Intraoperative euvolemia	All patients	All patients	All patients	All patients	Not reported	All patients
14. Preventing intraoperative hypothermia	Not reported	All patients	Not reported	All patients	Not reported	All patients
15. Minimally invasive surgery	Some patients	All patients	Not applicable	All patients	Not applicable	All patients
16. No peritoneal drainage	Some patients	Not reported	All patients	Some patients	Not reported	Not applicable
17. No nasogastric drainage	Some patients	Not reported	All patients	All patients	Not reported	Not reported
18. Postoperative analgesia	All patients	All patients	Not reported	All patients	All patients	All patients
19. Thromboprophylaxis	Not reported	All patients	Not reported	All patients	Not reported	All patients
20. Postoperative euvolemia	Not reported	Not reported	Not reported	Not reported	All patients	Not reported
21. Urinary catheterisation	All patients	All patients	Not reported	All patients	Not reported	All patients
22. Prevention of postoperative ileus	Not reported	All patients	Not reported	All patients	Not reported	Not applicable
23. Postoperative glycaemic control	Not reported	Not reported	Not reported	All patients	Not reported	Not reported
24. Postoperative nutritional care	All patients	All patients	All patients	All patients	All patients	All patients
25. Early mobilisation	Some patients	All patients	All patients	All patients	All patients	Not reported
Number of ERAS components applied	12	12	8	19	7	10

ERAS – enhanced recovery after surgery

*Based on 2018 ERAS protocol for elective colorectal surgery [9]. Interventions not applicable to craniotomies or stoma reversal procedures are indicated.

### Outcomes

The primary outcome in most studies was LOS, defined as the number of days postoperatively until discharge. Most studies reported mortality as a secondary outcome, with low rates across all studies (**Table 3**). Kurmi et al. [29] were only ones to use a standardised definition of complications (Clavien-Dindo), preventing meaningful grouping of this outcome in meta-analysis. Length of follow-up was 30 days for all studies, except for Gopakumar et al. [30], for whom it was three months, and Pirzada et al. [31], who did not report a follow-up length of time. Elayat et al. [32] (the only neurosurgical study) reported the highest mortality rate; two patients died in the ERAS group and three in the control group within 30 days of their operation. Three patients had a pulmonary embolism (PE) in the control group in Shetiwy et al. [28], with two resulting in death. Other adverse events included two anastomotic leaks (one in each group) in Nanavati et al. [27] and one anastomotic leak in the control group for Kurmi et al. [29].

**Table 3:** Study demographics and outcomes

Study	Group	Number of patients	Age in years, mean (SD)	Gender, n of females	Mortality, n	Length of stay, mean (SD)
Nanavati et al [27], 2014	Intervention	30	33.50 (12.36)	15	0	4.73 (1.34)
	Control	30	34.77 (14.40)	13	0	7.27 (1.36)
Shetiwy et al [28], 2017	Intervention	35	48.54 (12.29)	14	0	4.49 (0.853)
	Control	35	53.63 (11.5)	11	2	13.31 (6.897)
Kurmi et al [29], 2021	Intervention	15	39.42 (11.5)	3	0	1.58 (1.11)
	Control	15	41.42 (12.0)	5	0	6.58 (0.862)
Gopakumar et al [30], 2020	Intervention	78	Not reported	Not reported	Not reported	Not reported
	Control	78	Not reported	Not reported	Not reported	Not reported
Pirzada et al [31], 2017	Intervention	30	23.87 (4.56)	10	0	4.13 (1.04)
	Control	30	27.80 (9.99)	11	0	7.23 (1.16)
Elayat et al [32], 2021	Intervention	35	40.89 (13.61)	21	2	11.49 (9.04)
	Control	35	46.89 (13.95)	19	3	12.08 (8.76)

**Figure 2:**
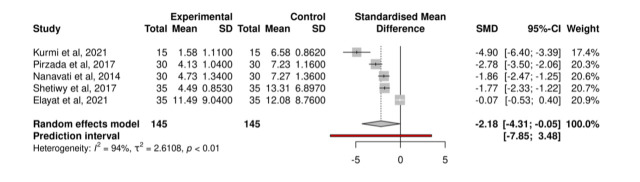
Forest plot of pooled length of stay analysis (measured in days).

The pooled analysis for LOS (**Figure 2**) showed reductions in the ERAS group with a standardised mean difference of -2.18 (95% confidence interval (CI) = -4.13, -.0.05; *P* < 0.01) days lower than the control group. However, the prediction interval was large (95% CI = -7.85, 3.48) and *I*^2^ was 94%, indicating substantial between-study heterogeneity [35]. A sensitivity analysis [36] found no outliers and there was no marked asymmetry in the funnel plot analysis (Figure S1 in the **Online Supplementary Document**), although Kurmi et al. [29] had a high effect size and high standard error. A sensitivity analysis excluding Elayat et al. [32] and including only general surgical patients showed similar results, with a standardised mean difference of -2.69 (95% CI = -4.86, -0.52; *P* < 0*.*01) and *I*^2^ of 84% (Figure S2 in the **Online Supplementary Document**). Due to high variability in reporting between studies and low study numbers, we did not conduct a meta-regression or pooled analysis of other outcomes.

A qualitative summary of the benefits and barriers to implementation of ERAS is summarised in Table S1 in the **Online Supplementary Document**.

## DISCUSSION

Our systematic review and meta-analysis showed that ERAS protocols reduced the length of hospital stay when implemented in LMICs. We observed high levels of between-study heterogeneity and variable uptake of ERAS components. For example, prehabilitation, preoperative optimisation, and management of anaemia were not reported by any studies.

The reduction in observed LOS is consistent with findings of a meta-analysis conducted in high-income settings [37], which found a reduction in LOS after colorectal surgery of 2.55 days, as compared to 2.18 in this analysis. Kurmi et al. [29] observed a larger LOS reduction (standardised mean error (SME) = -4.90; 95% CI = -6.40, -3.39) than other studies and was likely a significant contributor to heterogeneity. There are several potential explanations for this. For example, the study design had intervention patients cared for in a different surgical unit by a different surgeon and team than control patients. Furthermore, there was a lack of randomisation in most studies. Reduction in length of stay is considered unequivocally advantageous in high income settings, where there is a reliance on primary care and easy access to hospitals for identification and treatment of early postoperative complications. In LMICs, where healthcare infrastructure and distance from home is variable, there may need to be a more nuanced appreciation of the consequences of patients returning home soon after their surgery and how this can be safely facilitated.

Qualitatively, ERAS was perceived as improving outcomes and being economically beneficial for service provision by reducing LOS, yet most authors observed resistance to the introduction of a new protocol and a need for further education. While the application of the ERAS protocol was variable, prehabilitation and preoperative optimisation were consistently not undertaken across all studies. This finding was reiterated in a systematic review of Cochrane reviews, which found any pre-admission or pre-operative interventions to be exclusively studied in high-income countries [38]. Factors such as increased distance of the hospital from home may contribute to difficulty in patients presenting separately for prehabilitation prior to admission for surgery [12]. More fundamentally, this reflects differences in healthcare systems across income levels.

ERAS relies on the underlying healthcare system: it requires a multidisciplinary team and substantial nursing care, which may be challenging in LMICs with a high patient-to-nurse ratio [17]. It is underpinned by a continuous audit process requiring infrastructure that is commonly absent in LMICs and associated with high expenditure [39]. There are different stakeholders involved in lower-income settings. For instance, Elayat et al. [32] describe the role of relatives in postoperative patient feeding and mobilisation which would normally be undertaken by nurses or other support staff in high-income countries.

Resistance to change has also been consistently described as a barrier. Despite high-quality evidence, inertia is still present. This was a pattern observed in the introduction of the WHO Surgical Safety Checklist [40]. Applying individual ERAS protocol elements in the absence of stakeholder buy-in misses the opportunity for an enhanced perioperative care environment delivered by an integrated multidisciplinary team [41]. The utilisation of implementation science strategies [42] is essential in improving the uptake of ERAS protocols through a better understanding of the real-world challenges faced in LMIC settings. Importantly, this will only lead to limited benefits in the absence of strategies to strengthen surgical care systems [43].

There are several limitations to this analysis. The studies included have a relatively high risk of bias and substantial between-study heterogeneity, so the results of the pooled analysis should be interpreted with caution. Heterogeneity was likely further increased by grouping studies evaluating different surgical specialities; ideally, our analysis would have focused on similar procedures and patient populations, but was limited by numbers (although our sensitivity analysis excluding neurosurgical patients showed similar results). While LOS is widely used as an endpoint in ERAS literature, is an imperfect outcome measure and could be influenced by confounding variables beyond the intervention. Furthermore, most studies were not RCTs and all were conducted in single centres with small sample sizes, limiting the ability to address confounding and undertake meaningful meta-regression analyses. Studies failed to provide missing data at the level of each ERAS component or details on the number of patients receiving each component, which made it difficult to ascertain which components influence outcomes. Extensive exclusion criteria employed by most studies means the sample in this analysis is likely different to the target population in LMICs, introducing an added layer of uncertainty around the real-world effectiveness of the intervention. Additionally, LMICs are not a homogenous population; there are significant between and within countries which limit generalisability.

All studies in this analysis excluded either elderly patients or those with low functional status or uncontrolled comorbid conditions (**Table 1**). There are no recommendations to exclude patients from ERAS protocols on these criteria, and ERAS protocols have been shown to be as effective in elderly patients with comorbidities as in younger patients despite potential differences in the number of ERAS components adhered to [44]. Nanavati et al. [27] and Pirzada et al. [31] both reported particularly low mean ages (23.87 (standard deviation (SD) = 4.56) vs. 33.50 (SD = 12.36)) for the ERAS group, which are not fully explained by the exclusion criteria. This may reflect the surgical population in LMICs being inherently different, which is consistent with literature describing surgical patients in lower-income settings as often being younger and presenting with different disease aetiologies [19].

These limitations have likely contributed to the exclusion of LMIC studies from most meta-analyses conducted on the effectiveness of ERAS protocols and other perioperative interventions, leading to a lack of high-level evidence on the effectiveness of these interventions in LMIC settings. Future studies should focus on multicentre data entry into centralised databases which enable compliance with individual elements of the protocol to be determined and multivariable analysis to be undertaken. There is also a need for qualitative analysis of barriers to implementation to improve the understanding of the perioperative environment in lower-income settings, which could help in formulating future LMIC-specific guidelines.

## CONCLUSIONS

Despite high levels of between-study heterogeneity, we found that implementation of ERAS protocols is feasible in LMICs, with implications for reduced length of hospital stay as a meaningful outcome. High-quality data on perioperative practices in LMICs and supplementary qualitative analysis are needed to further understand barriers to perioperative optimisation in LMIC healthcare settings.

## Additional material


Online Supplementary Document
