# Supplementary material for: Perioperative optimisation in low- and middle-income countries (LMICs): A systematic review and meta-analysis of enhanced recovery after surgery (ERAS)
Source: J Glob Health. 2023 Oct 3;13:04114. doi: 10.7189/jogh.13.04114 (PMC10546475; doi:10.7189/jogh.13.04114)
Supplement: Online Supplementary Document [file jogh-13-04114-s001.pdf]

Supplementary Figures

<u>Supplementary Figure 1:</u> Pooled Length of Stay Analysis – Funnel Plot

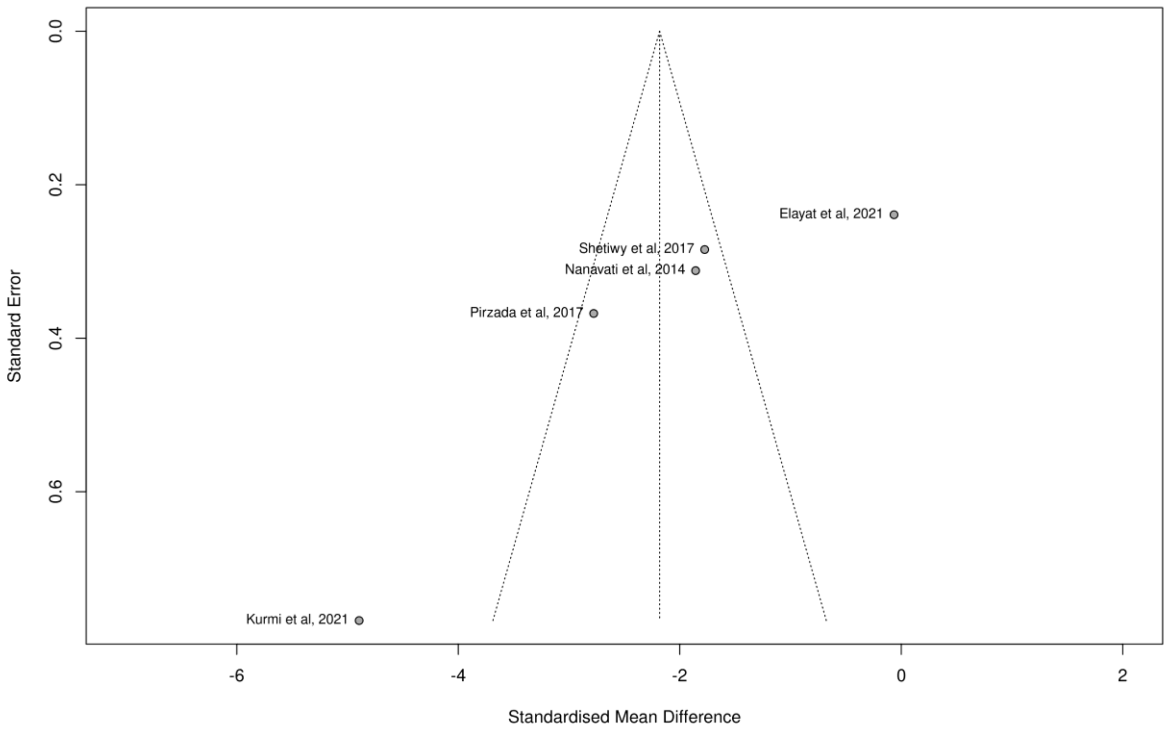

<u>Supplementary Figure 2:</u> Sensitivity Analysis Excluding Elayat et Al – Forrest Plot

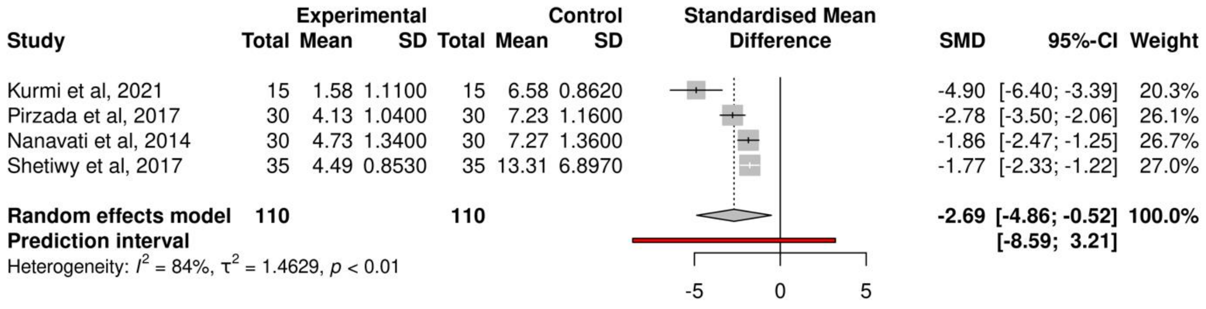

<u>Supplementary Table 1:</u> Perceived Benefits, Barriers and Adaptations for ERAS Implementation in LMICs

Study	Benefits	Barriers
Nanavati *et al*, 2014	Facilitates early discharge which saves considerable healthcare costs for hospital and patient<br>Increased patient turnover	An 'inertia' seen amongst surgeons in departing from traditional methods<br>Variability in protocol application<br>Restricted access to laparoscopic surgery and resources in general in government hospitals
Shetiwy *et al*, 2017	Reduced length of stay<br>Reduced complications<br>Earlier gut function restoration<br>No effect on readmission or mortality	Not reported
Kurmi *et al*, 2021	Decreased in hospital stay and facilitated day-case stoma closure<br>No change in postoperative pain	Not reported
Gopakumar *et al*, 2020	Reduced length of stay and complications<br>Higher patient satisfaction	Need for increased education of multi-disciplinary team
Pirzada *et al*, 2017	Reduced length of stay<br>Reduced pressure on public hospitals	Not reported
Elayat *et al*, 2021	Reduced intensive care unit (ICU) stay<br>Decreased pain and opioid requirement after surgery<br>Better postoperative glycaemic control<br>Earlier postoperative mobilization	Need buy in from relatives who play a large part in postoperative care due to cultural values<br>Need to combat "ignorance, fear and hesitation"<br>Limited post-discharge care

Appendices

Appendix 1: Full search criteria

prehab* or p?operative optimi?ation or p?operative exercise or (p?operative and (risk adj5 reduc*)) or p?operative nutrition or p?operative training or p?operative inspiratory muscle training or "enhanced recovery after surgery" or eras

Only applied to MEDLINE + PubMed as not LMIC specific databases:

AND afghanistan[MeSH] OR albania[MeSH] OR algeria[MeSH] OR american samoa[MeSH] OR angola[MeSH] OR antigua and barbuda[MeSH] OR argentina[MeSH] OR armenia[MeSH] OR aruba[MeSH] OR azerbaijan[MeSH] OR bahrain[MeSH] OR bangladesh[MeSH] OR barbados[MeSH] OR republic of belarus[MeSH] OR belize[MeSH] OR benin[MeSH] OR bhutan[MeSH] OR bolivia[MeSH] OR bosnia and herzegovina[MeSH] OR botswana[MeSH] OR brazil[MeSH] OR bulgaria[MeSH] OR burkina faso[MeSH] OR burundi[MeSH] OR cabo verde[MeSH] OR cambodia[MeSH] OR cameroon[MeSH] OR central african republic[MeSH] OR chad[MeSH] OR chile[MeSH] OR china[MeSH] OR colombia[MeSH] OR comoros[MeSH] OR democratic republic of the congo[MeSH] OR congo[MeSH] OR costa rica[MeSH] OR cote d'ivoire[MeSH] OR croatia[MeSH] OR cuba[MeSH] OR cyprus[MeSH] OR czech republic[MeSH] OR djibouti[MeSH] OR dominica[MeSH] OR dominican republic[MeSH] OR ecuador[MeSH] OR egypt[MeSH] OR el salvador[MeSH] OR equatorial guinea[MeSH] OR eritrea[MeSH] OR estonia[MeSH] OR swaziland[MeSH] OR ethiopia[MeSH] OR fiji[MeSH] OR gabon[MeSH] OR gambia[MeSH] OR georgia (republic)[MeSH] OR ghana[MeSH] OR gibraltar[MeSH] OR greece[MeSH] OR grenada[MeSH] OR guam[MeSH] OR guatemala[MeSH] OR guinea[MeSH] OR guinea bissau[MeSH] OR guyana[MeSH] OR haiti[MeSH] OR honduras[MeSH] OR hungary[MeSH] OR india[MeSH] OR indonesia[MeSH] OR iran[MeSH] OR iraq[MeSH] OR jamaica[MeSH] OR jordan[MeSH] OR kazakhstan[MeSH] OR kenya[MeSH] OR democratic people's republic of korea[MeSH] OR republic of korea[MeSH] OR kosovo[MeSH] OR kyrgyzstan[MeSH] OR laos[MeSH] OR latvia[MeSH] OR lebanon[MeSH] OR lesotho[MeSH] OR liberia[MeSH] OR libya[MeSH] OR lithuania[MeSH] OR macau[MeSH] OR republic of north macedonia[MeSH] OR madagascar[MeSH] OR malawi[MeSH] OR malaysia[MeSH] OR indian ocean islands[MeSH] OR mali[MeSH] OR malta[MeSH] OR micronesia[MeSH] OR palau[MeSH] OR mauritania[MeSH] OR mauritius[MeSH] OR mexico[MeSH] OR moldova[MeSH] OR mongolia[MeSH] OR montenegro[MeSH] OR morocco[MeSH] OR mozambique[MeSH] OR myanmar[MeSH] OR namibia[MeSH] OR nepal[MeSH] OR netherlands antilles[MeSH] OR nicaragua[MeSH] OR niger[MeSH] OR nigeria[MeSH] OR oman[MeSH] OR pakistan[MeSH] OR panama[MeSH] OR papua new guinea[MeSH] OR paraguay[MeSH] OR peru[MeSH] OR philippines[MeSH] OR poland[MeSH] OR portugal[MeSH] OR puerto rico[MeSH] OR romania[MeSH] OR russia[MeSH] OR rwanda[MeSH] OR samoa[MeSH] OR sao tome and principe[MeSH] OR saudi arabia[MeSH] OR senegal[MeSH] OR serbia[MeSH] OR seychelles[MeSH] OR sierra leone[MeSH] OR slovakia[MeSH] OR slovenia[MeSH] OR melanesia[MeSH] OR somalia[MeSH] OR south africa[MeSH] OR south sudan[MeSH] OR sri lanka[MeSH] OR saint kitts and nevis[MeSH] OR saint lucia[MeSH] OR saint vincent and the grenadines[MeSH] OR sudan[MeSH] OR suriname[MeSH] OR syria[MeSH] OR tajikistan[MeSH] OR tanzania[MeSH] OR thailand[MeSH] OR timor leste[MeSH] OR togo[MeSH] OR tonga[MeSH] OR trinidad and tobago[MeSH] OR tunisia[MeSH] OR turkey[MeSH] OR turkmenistan[MeSH] OR

uganda[MeSH] OR ukraine[MeSH] OR uruguay[MeSH] OR uzbekistan[MeSH] OR vanuatu[MeSH] OR venezuela[MeSH] OR vietnam[MeSH] OR gaza[MeSH] OR tuvalu[MeSH] OR Solomon islands[MeSH] OR palau[MeSH] OR northern mariana islands[MeSH] OR north Macedonia[MeSH] OR nauru[MeSH] OR micronesia[MeSH] OR marshall islands[MeSH] OR maldives[MeSH] OR kiribati[MeSH] OR eswatini[MeSH] OR middle east[MeSH] OR yemen[MeSH] OR yugoslavia[MeSH] OR zambia[MeSH] OR zimbabwe[MeSH] OR africa south of the sahara[MeSH] OR africa, central[MeSH] OR africa, northern[MeSH] OR africa, southern[MeSH] OR africa, eastern[MeSH] OR africa, western[MeSH] OR west indies[MeSH] OR indian ocean islands[MeSH] OR caribbean region[MeSH] OR central america[MeSH] OR latin america[MeSH] OR south america[MeSH] OR asia, central[MeSH] OR asia, northern[MeSH] OR asia, southeastern[MeSH] OR asia, western[MeSH] OR europe, eastern[MeSH] OR developing countries[MeSH] OR afghanistan[Text Word] OR albania[Text Word] OR algeria[Text Word] OR american samoa[Text Word] OR angola[Text Word] OR antigua[Text Word] OR barbuda[Text Word] OR argentina[Text Word] OR armenia[Text Word] OR armenian[Text Word] OR aruba[Text Word] OR azerbaijan[Text Word] OR bahrain[Text Word] OR bangladesh[Text Word] OR barbados[Text Word] OR belarus[Text Word] OR byelarus[Text Word] OR belorussia[Text Word] OR byelorussian[Text Word] OR belize[Text Word] OR british honduras[Text Word] OR benin[Text Word] OR dahomey[Text Word] OR bhutan[Text Word] OR bolivia[Text Word] OR bosnia[Text Word] OR herzegovina[Text Word] OR botswana[Text Word] OR bechuanaland[Text Word] OR brazil[Text Word] OR brasil[Text Word] OR bulgaria[Text Word] OR burkina faso[Text Word] OR burkina fasso[Text Word] OR upper volta[Text Word] OR burundi[Text Word] OR urundi[Text Word] OR cabo verde[Text Word] OR cape verde[Text Word] OR cambodia[Text Word] OR kampuchea[Text Word] OR khmer republic[Text Word] OR cameroon[Text Word] OR cameron[Text Word] OR cameroun[Text Word] OR central african republic[Text Word] OR ubangi shari[Text Word] OR chad[Text Word] OR chile[Text Word] OR china[Text Word] OR colombia[Text Word] OR comoros[Text Word] OR comoro islands[Text Word] OR mayotte[Text Word] OR congo[Text Word] OR zaire[Text Word] OR costa rica[Text Word] OR cote d'ivoire[Text Word] OR cote d' ivoire[Text Word] OR cote divoire[Text Word] OR cote d ivoire[Text Word] OR ivory coast[Text Word] OR croatia[Text Word] OR cuba[Text Word] OR cyprus[Text Word] OR czech republic[Text Word] OR czechoslovakia[Text Word] OR djibouti[Text Word] OR french somaliland[Text Word] OR dominica[Text Word] OR dominican republic[Text Word] OR ecuador[Text Word] OR egypt[Text Word] OR united arab republic[Text Word] OR el salvador[Text Word] OR equatorial guinea[Text Word] OR spanish guinea[Text Word] OR eritrea[Text Word] OR estonia[Text Word] OR eswatini[Text Word] OR swaziland[Text Word] OR ethiopia[Text Word] OR fiji[Text Word] OR gabon[Text Word] OR gabonese republic[Text Word] OR gambia[Text Word] OR georgia[Text Word] OR georgian[Text Word] OR ghana[Text Word] OR gold coast[Text Word] OR gibraltar[Text Word] OR greece[Text Word] OR grenada[Text Word] OR guam[Text Word] OR guatemala[Text Word] OR guinea[Text Word] OR guyana[Text Word] OR guiana[Text Word] OR haiti[Text Word] OR hispaniola[Text Word] OR honduras[Text Word] OR hungary[Text Word] OR india[Text Word] OR indonesia[Text Word] OR timor[Text Word] OR iran[Text Word] OR iraq[Text Word] OR isle of man[Text Word] OR jamaica[Text Word] OR jordan[Text Word] OR kazakhstan[Text Word] OR kazakh[Text Word] OR kenya[Text Word] OR korea[Text Word] OR kosovo[Text Word] OR kyrgyzstan[Text Word] OR kirghizia[Text Word] OR kirgizstan[Text Word] OR kyrgyz republic[Text Word] OR kirghiz[Text Word] OR laos[Text Word] OR lao pdr[Text Word] OR lao people's democratic republic[Text Word] OR latvia[Text Word] OR lebanon[Text Word] OR lesotho[Text Word] OR basutoland[Text Word] OR liberia[Text Word] OR

libya[Text Word] OR libyan arab jamahiriya[Text Word] OR lithuania[Text Word] OR macau[Text Word] OR macao[Text Word] OR macedonia[Text Word] OR madagascar[Text Word] OR malagasy republic[Text Word] OR malawi[Text Word] OR nyasaland[Text Word] OR malaysia[Text Word] OR maldives[Text Word] OR indian ocean[Text Word] OR mali[Text Word] OR malta[Text Word] OR micronesia[Text Word] OR kiribati[Text Word] OR marshall islands[Text Word] OR nauru[Text Word] OR northern mariana islands[Text Word] OR palau[Text Word] OR tuvalu[Text Word] OR mauritania[Text Word] OR mauritius[Text Word] OR mexico[Text Word] OR moldova[Text Word] OR moldovian[Text Word] OR mongolia[Text Word] OR montenegro[Text Word] OR morocco[Text Word] OR ifni[Text Word] OR mozambique[Text Word] OR portuguese east africa[Text Word] OR myanmar[Text Word] OR burma[Text Word] OR namibia[Text Word] OR nepal[Text Word] OR netherlands antilles[Text Word] OR nicaragua[Text Word] OR niger[Text Word] OR nigeria[Text Word] OR oman[Text Word] OR muscat[Text Word] OR pakistan[Text Word] OR panama[Text Word] OR papua new guinea[Text Word] OR paraguay[Text Word] OR peru[Text Word] OR philippines[Text Word] OR philipines[Text Word] OR phillipines[Text Word] OR phillippines[Text Word] OR poland[Text Word] OR polish people's republic[Text Word] OR portugal[Text Word] OR portuguese republic[Text Word] OR puerto rico[Text Word] OR romania[Text Word] OR russia[Text Word] OR russian federation[Text Word] OR ussr[Text Word] OR soviet union[Text Word] OR union of soviet socialist republics[Text Word] OR rwanda[Text Word] OR ruanda[Text Word] OR samoa[Text Word] OR pacific islands[Text Word] OR polynesia[Text Word] OR samoan islands[Text Word] OR sao tome and principe[Text Word] OR saudi arabia[Text Word] OR senegal[Text Word] OR serbia[Text Word] OR seychelles[Text Word] OR sierra leone[Text Word] OR slovakia[Text Word] OR slovak republic[Text Word] OR slovenia[Text Word] OR melanesia[Text Word] OR solomon island[Text Word] OR solomon islands[Text Word] OR norfolk island[Text Word] OR somalia[Text Word] OR south africa[Text Word] OR south sudan[Text Word] OR sri lanka[Text Word] OR ceylon[Text Word] OR saint kitts and nevis[Text Word] OR st kitts and nevis[Text Word] OR saint lucia[Text Word] OR st lucia[Text Word] OR saint vincent[Text Word] OR st vincent[Text Word] OR grenadines[Text Word] OR sudan[Text Word] OR suriname[Text Word] OR surinam[Text Word] OR syria[Text Word] OR syrian arab republic[Text Word] OR tajikistan[Text Word] OR tadjikistan[Text Word] OR tadzhikistan[Text Word] OR tadzhik[Text Word] OR tanzania[Text Word] OR tanganyika[Text Word] OR thailand[Text Word] OR siam[Text Word] OR timor leste[Text Word] OR east timor[Text Word] OR togo[Text Word] OR togolese republic[Text Word] OR tonga[Text Word] OR trinidad[Text Word] OR tobago[Text Word] OR tunisia[Text Word] OR turkey[Text Word] OR turkmenistan[Text Word] OR turkmen[Text Word] OR uganda[Text Word] OR ukraine[Text Word] OR uruguay[Text Word] OR uzbekistan[Text Word] OR uzbek[Text Word] OR vanuatu[Text Word] OR new hebrides[Text Word] OR venezuela[Text Word] OR vietnam[Text Word] OR viet nam[Text Word] OR middle east[Text Word] OR west bank[Text Word] OR gaza[Text Word] OR palestine[Text Word] OR yemen[Text Word] OR yugoslavia[Text Word] OR zambia[Text Word] OR zimbabwe[Text Word] OR northern rhodesia[Text Word] OR global south[Text Word] OR africa south of the sahara[Text Word] OR sub saharan africa[Text Word] OR subsaharan africa[Text Word] OR central africa[Text Word] OR north africa[Text Word] OR northern africa[Text Word] OR magreb[Text Word] OR maghrib[Text Word] OR sahara[Text Word] OR southern africa[Text Word] OR east africa[Text Word] OR eastern africa[Text Word] OR west africa[Text Word] OR western africa[Text Word] OR west indies[Text Word] OR indian ocean islands[Text Word] OR caribbean[Text Word] OR central america[Text Word] OR latin america[Text Word] OR south america[Text Word] OR central asia[Text Word] OR north asia[Text Word] OR northern asia[Text Word] OR southeastern asia[Text Word] OR

south eastern asia[Text Word] OR southeast asia[Text Word] OR south east asia[Text Word] OR western asia[Text Word] OR east europe[Text Word] OR eastern europe[Text Word] OR developing country[Text Word] OR developing countries[Text Word] OR developing nation[Text Word] OR developing nations[Text Word] OR developing population[Text Word] OR developing populations[Text Word] OR developing world[Text Word] OR less developed country[Text Word] OR less developed countries[Text Word] OR less developed nation[Text Word] OR less developed nations[Text Word] OR less developed world[Text Word] OR lesser developed countries[Text Word] OR lesser developed nations[Text Word] OR under developed country[Text Word] OR under developed countries[Text Word] OR under developed nations[Text Word] OR under developed world[Text Word] OR underdeveloped country[Text Word] OR underdeveloped countries[Text Word] OR underdeveloped nation[Text Word] OR underdeveloped nations[Text Word] OR underdeveloped population[Text Word] OR underdeveloped populations[Text Word] OR underdeveloped world[Text Word] OR middle income country[Text Word] OR middle income countries[Text Word] OR middle income nation[Text Word] OR middle income nations[Text Word] OR middle income population[Text Word] OR middle income populations[Text Word] OR low income country[Text Word] OR low income countries[Text Word] OR low income nation[Text Word] OR low income nations[Text Word] OR low income population[Text Word] OR low income populations[Text Word] OR lower income country[Text Word] OR lower income countries[Text Word] OR lower income nations[Text Word] OR lower income population[Text Word] OR lower income populations[Text Word] OR underserved countries[Text Word] OR underserved nations[Text Word] OR underserved population[Text Word] OR underserved populations[Text Word] OR under served population[Text Word] OR under served populations[Text Word] OR deprived countries[Text Word] OR deprived population[Text Word] OR deprived populations[Text Word] OR poor country[Text Word] OR poor countries[Text Word] OR poor nation[Text Word] OR poor nations[Text Word] OR poor population[Text Word] OR poor populations[Text Word] OR poor world[Text Word] OR poorer countries[Text Word] OR poorer nations[Text Word] OR poorer population[Text Word] OR poorer populations[Text Word] OR developing economy[Text Word] OR developing economies[Text Word] OR less developed economy[Text Word] OR less developed economies[Text Word] OR underdeveloped economies[Text Word] OR middle income economy[Text Word] OR middle income economies[Text Word] OR low income economy[Text Word] OR low income economies[Text Word] OR lower income economies[Text Word] OR low gdp[Text Word] OR low gnp[Text Word] OR low gross domestic[Text Word] OR low gross national[Text Word] OR lower gdp[Text Word] OR lower gross domestic[Text Word] OR lmic[Text Word] OR lmics[Text Word] OR third world[Text Word] OR lami country[Text Word] OR lami countries[Text Word] OR transitional country[Text Word] OR transitional countries[Text Word] OR emerging economies[Text Word] OR emerging nation[Text Word] OR emerging nations[Text Word]

Appendix 2: Data extraction form

General information

Study ID
Title
Title of paper / abstract / report that data are extracted from
Authors
Journal
Publication Year
Country
Country income level at time of study
Use historical world bank country income level classification to determine the level at the year of publication - found here https://datahelpdesk.worldbank.org/knowledgebase/articles/906519-world-bank-country-and-lending-groups

1. Lower middle income
2. Low income

Speciality
Notes

Characteristics of included studies

Methods

Aim of study
Study design

1. Randomised controlled trial
2. Non-randomised experimental study
3. Cohort study
4. Cross sectional study
5. Case control study
6. Systematic review
7. Qualitative research
8. Prevalence study
9. Case series
10. Case report
11. Diagnostic test accuracy study
12. Clinical prediction rule
13. Economic evaluation
14. Text and opinion
15. Other

Start date
End date
Study funding sources
Possible conflicts of interest for study authors
Comparison Group

1. Routine care
2. Other intervention (if so specify intervention in notes)

Participants

Cancer Patients?

1. Yes, all
2. Yes, some

3. No

Inclusion criteria
Exclusion criteria
Method of recruitment of participants

1. Phone
2. Mail
3. Clinic patients
4. Voluntary
5. Other

Total number of participants
Participants in Intervention Group
Participants in Control Group

Intervention

Intervention
Describe the specific intervention - ERAS, prehabilitation etc
Is this a validated intervention?
If ERAS then yes, otherwise is there evidence of this being a validated intervention mentioned in the paper/found elsewhere

1. Yes
2. No

If the intervention is ERAS which components are being applied? Tick all which apply
Components of ERAS described here https://www.ncbi.nlm.nih.gov/pmc/articles/PMC3202008/

1. Preoperative components
2. Intraoperative components
3. Postoperative components
4. Not ERAS so NA

Please specify which ERAS components are being applied
Select all the perioperative intervention types that have been included in this study
Please differentiate between when patients were offered counselling on interventions (eg exercise/diet/smoking cessation) and whether patients actually completed the intervention and that compliance was measured

1. Exercise
2. Nutrition
3. Psychological
4. Smoking cessation
5. Alcohol cessation
6. Advice on nutrition
7. Advice on exercise
8. Advice on smoking cessation
9. Advice on alcohol cessation

Duration of intervention
Please specify how long before AND after the operation the intervention was applied for
Setting for Intervention

1. Hospital
2. Home
3. Outpatient appointments
4. Other

Care provider leading intervention
Please tick all who apply

1. Surgeon
2. Anaesthetist
3. Nurse
4. Physiotherapist
5. Dietician
6. Psychologist

7. Patient-led
8. Other - please specify in notes

If exercise intervention, please specify type and frequency of exercise
If nutrition intervention, please specify contents of supplement and route of administration
If psychological intervention , please specify details of intervention
If nutritional status was measured, what marker was used for nutrition?
Please specify all used including BMI, percentage weight loss, albumin etc
What training, if any, was provided to practitioners delivering the intervention?
How was compliance with the intervention ensured?

Feasibility

Cost-effectiveness
For this section please include any free text mentioned on the following themes or if they were formally assessed in the study
Perceived benefits of intervention
Perceived barriers to implementing intervention
Adaptation of intervention for low resource environment
Did delivery of intervention go as planned? What issues were faced?
Adverse events?
Key conclusions
Other points of interest

Outcomes

Primary outcome
Other outcomes measured - please include all
Was quality of life measured?
If complications were measures how were they defined?
Give details of the complications classification system used eg: Clavien Dindo
Length of follow up (days)
Number of patients lost to follow-up
Time outcomes were measured
Please specify if outcomes were measured at different points after the operation.
Method of follow-up

1. Telephone
2. Patient survey
3. Electronic records
4. Not reported
5. Other

Results

Baseline Population Characteristics
If any item is not available please enter NA.

	Intervention	Control	Overall
**Age - average**			
**Sex - number female**			
**BMI - average**			

	Intervention	Control	Overall
**Smoking - number of smokers**			
**Cardiovascular comorbidity - number of patients**			
**Surgical approach - number of patients undergoing open operation**			
**ASA (number of patients in each grade)**			

Method of aggregation
Please specify how results were aggregated if not mean and SD.

Outcome table (Mortality)

	Mean	SD	Number of Patients
**Intervention**			
**Control**			

Outcome table (All Complications)

	Mean	SD	Number of Patients
**Intervention**			
**Control**			

Outcome table (Major Complications)

	Mean	SD	Number of Patients
**Intervention**			
**Control**			

Outcome table (Surgical Site Infection)

	Mean	SD	Number of Patients
**Intervention**			
**Control**			

Outcome table (Length of Stay)

	Mean	SD	Number of Patients
**Intervention**			
**Control**			

<u>Appendix 3:</u> Quality Assessment Components

<u>Appendix 3A:</u> Newcastle Ottawa Scale

Study	Nanavati *et al*, 2014	Kurmi *et al*, 2021	Gopakumar el al, 2020	Elayat *et al*, 2021
Total Score	5	4	3	6
Representativeness of exposed cohort	Selected group of users	Selected group of users	Selected group of users	Selected group of users
Selection of non-exposed cohort	Drawn from same community as exposed cohort	No description of derivation of non-exposed cohort	Drawn from same community as exposed cohort	Drawn from same community as exposed cohort
Ascertainment of exposure	Secure record	No description	No description	Secure record
Demonstration that outcome of interest was not present at start of study	Yes	Yes	Yes	Yes
Comparability of cohorts on the basis of design or analysis	No	No	No	No
Assessment of outcome	No description	Independent blind assessment	No description	Independent blind assessment
Was follow up long enough for outcomes to occur?	Yes (30 days or more)	Yes (30 days or more)	Yes (30 days or more)	No (Under 30 days)
Adequacy of follow up cohorts	Complete follow up – all subjects accounted for	Complete follow up – all subjects accounted for	No statement	Complete follow up – all subjects accounted for

<u>Appendix 3B:</u> Cochrane Risk of Bias Tool

Study	Shetiwy *et al*, 2017	Pirzada *et al*, 2017
Overall risk of bias judgement	Some concerns	Some concerns
Overall predicted direction of bias	Favours experimental	Favours experimental
Randomisation process	Some concerns	Some concerns
Effect of assignment to intervention	Some concerns	Some concerns
Effect of adhering to intervention	Low risk	Low risk
Measurement of outcome	Some concerns	Low risk
Selection of reported result	Some concerns	Low risk
